# Supplementary material for: Tuberculosis Preventive Treatment in People Living with HIV in Uganda: Facilitators and Barriers for Initiation and Completion
Source: Trop Med Infect Dis. 2025 Oct 27;10(11):303. doi: 10.3390/tropicalmed10110303 (PMC12656017; doi:10.3390/tropicalmed10110303)
Supplement: Supplementary file 1 [file tropicalmed-10-00303-s001.zip › tropicalmed-3943337-supplementary.pdf]

Supplementary Table S1: Facilitators for TPT initiation among PLHIV in selected health facilities of Uganda during 2022-2024

Subtheme	Description	Quote
**Patient level facilitators for TPT Initiation**	These are individual factors that enable and support patients to successfully start TPT. These facilitators promote readiness, willingness, and ability to initiate TPT among PLHIV.	
Positive feedback about TPT from peers	The PLHIV felt that hearing positive feedback from peers motivated them to initiate TPT. The majority of the PLHIV interviewed mentioned that they would recommend TPT to their peers.	When asked if they would recommend TPT, Patient 04 said: *"Yes, I would advise them to come and take TPT in order for them not get TB in case they encounter someone with TB."*
Perceived benefits of TPT	According to PLHIV, perceived benefits of TPT motivate them to initiate and adhere to the treatment course. The benefits mentioned included; reduced risk of TB, enhanced quality of life and improved health.	Patient 01 mentioned that: *"It will help me because in the community where I live, many are sick with TB so it will help me not to get the disease."*<br><br>Patient 02 said: *"Yes, it helps to feel alive, in case when TB comes it doesn't mistreat me like a person who didn't get TPT."*
Trust in HCWs and their prescriptions	Patients are more likely to initiate TPT when they trust the HCWs due to the health education provided. The patients feel confident in the HCWs' expertise and knowledge and believe in the efficacy of TPT.	When asked if there were any risks, HCW 01 said:<br><br>*"The people who gave me these medicines are trained doctors and my heart was contended"*
**Provider level facilitators for TPT Initiation**	These are factors related to healthcare providers that enable and support patients to successfully start TPT.	
HCWs initiating TPT in community	HCWs believe that community programs are efficient for TPT initiation especially among PLHIV who are unable to visit the HFs.	HCW 02 said:<br><br>*"We also have community TPT initiation. We go to the community and initiate eligible persons on TPT."*

Pro-activeness of HCWs to flag eligible patients	As a practice at majority of the HFs, HCWs conduct file audits to identify PLHIV who are eligible but have never been initiated on TPT.	HCW 03 said: *"We flag files for all patients that are due for TPT initiation. So, the next time they visit the health facility, whichever HCW sees the patient will initiate them on TPT."*
Awareness among HCWs about TPT	HCWs have the knowledge on TPT eligibility criteria which facilitates timely initiation. Relatedly, HCWs conduct routine health education for PLHIV to inform them on the benefits of TPT.	HCW 04 explained that: *"The information we give to these clients when we are counselling and preparing them for TPT is important so that they do not refuse treatment. We have also had some CMEs for HCWs about the importance of TPT for our clients so that the HCWs are competent enough to educate our clients and prepare them well for TPT."*
HCWs prioritizing TB prevention over treatment	HCWs believe that TPT is a cost-effective strategy for reducing TB related morbidity and mortality.	*"TPT prevents patients from getting TB disease. The logistics we use on a patient to prevent them from getting TB cannot be the same as the ones used for treating TB. So, the prevention measures should be emphasized."*
**System related facilitators for TPT Initiation**	These are organizational, structural, and policy-related factors that enable and support healthcare providers and patients to successfully initiate TPT.	
Availability of shorter regimens	The introduction of 3HP and 1HP has improved the initiation rates and acceptability due to the shorter duration as compared to 6H.	HCW 05 explained that pill burden is one of the challenges to TPT initiation, however, with the introduction of these shorter regimens, TPT is now more acceptable to PLHIV. *"We have a variety of TPT regimens which have helped solve the challenge of pill burden"*
Availability of printed operational guidelines on TPT at health facilities	The availability of SOPs on TPT initiation, eligibility criteria and dosing facilitate timely initiation of PLHIV on TPT.	*"There are also guidelines in the HIV/TB clinic on the dosing, eligibility of TPT."*

Establishing community-based services for facilitating TPT initiation	Community programs are efficient for TPT initiation especially among PLHIV with time and financial constraints.	*“HCWs are provided with transport to deliver drugs to patients in the community when the patients are unable to visit the HF especially because of the current situation we are facing.”*
Integration of TPT in HIV diagnostic and care provision	Aligning TPT and ART dispensing schedules increases demand and uptake of TPT and ensures timely initiation of TPT among PLHIV.	*“Implementation of TPT at this facility has been well integrated into our HIV services. We are following the HIV 2020 guidelines as per now to implement TPT among our people living with HIV.”*

Supplementary Table S2: Barriers for TPT initiation among PLHIV in selected health facilities of Uganda during 2022-2024

**Patient level barriers for TPT Initiation**	These are individual factors that hinder/delay TPT initiation among eligible PLHIV.	
Fear of TPT side effects	The fear of drug-related side effects was reported as a key barrier to starting TPT. Participants expressed their fear of taking TPT treatment for fear of side effects.	HCW 06 mentioned that:<br><br>*"Majority of the PLHIV fear drug side effects. People ask 'Doctor, will it not bring me side effects when am also taking these ARVs?'"*
Lack of awareness on TPT benefits	Although patients are routinely educated on the benefits of TPT, there are some that are hesitant/adamant to take the drugs.	HCW 02 reported that:<br><br>*"I think the biggest challenge is that people do not know why they are taking the drug. Someone feels okay, they don't feel sick, they're wondering 'why am I taking these drugs?' They might accept the TPT medicines when they are given to them but when they reach home, they will not swallow them."*
Pill burden	Some PLHIV are hesitant to initiate TPT due to pill burden, especially those that are receiving treatment for other comorbidities such as hypertension, diabetes, among others.	HCW 07 reported information from a patient:<br><br>*"Doctor, I have enough drugs". At some point, even me as a service provider, you see the patient is on ARVs and is not on a fixed drug combination, and has other comorbidities, the NCDs, is on treatment for hypertension and diabetes."*
Financial and time constraints for health facility visits (Proxy visits for refills)	HCWs reported that there are patients who are represented at clinical appointments, they send their relative/spouse to get drug refills for them. When they are not evaluated, they cannot be initiated on TPT.	*"Long distances to the HF which poses a challenge in the linkage process. Some patients may be willing to be initiated on TPT but are unable to visit the HF due to long distances and lack of transport."*
**Provider level barriers for TPT Initiation**	These are challenges related to healthcare providers that hinder/delay TPT initiation among eligible PLHIV.	

Heavy workload for HCWs	Understaffing emerged as one of the challenges for TPT initiation. HCWs have a heavy workload which hinders timely TPT initiation.	*"Long procedures of TPT initiation coupled with insufficient time for screening and offering health education. You find only 1 HCW at health facilities where one HCW has to attend to over 300 patients every day."*
Knowledge gaps among HCWs	Some HCWs lack the knowledge to identify clients eligible for TPT which delays initiation.	*"There are some HCWs that may not know the guidelines on TPT eligibility and initiation and this may delay the process of initiating eligible patients on TPT."*
Hesitancy due to pill burden	Some HCWs reported that they were hesitant to initiate some patients on TPT due to the pill burden. Based on the HCW's judgement on the ability of a patient to accept and adhere to treatment, TPT initiation is delayed.	*"There are some patients with a high pill burden, someone is taking ARVs, anti-hypertensive drugs, is also on medication for diabetes. It becomes difficult to even convince this patient to start on another set of drugs."*
**System related barriers for TPT Initiation**	These are organizational, structural, and policy-related challenges that hinder healthcare providers and patients to successfully initiate TPT.	
Difficulty to rule out TB among children	Children with TB may exhibit non-specific symptoms like fever, cough, and weight loss, making diagnosis difficult.<br>Relatedly, sputum production is difficult among children, making it hard to diagnose TB using sputum-based tests.	HCW 08 mentioned that:<br><br>*"Most children persistently have cough which makes them ineligible for TPT initiation. If you are not keen/careful, you may never initiate the child on TPT. Every time the child visits the HF, they are coughing."*
Requirement of baseline blood investigations before starting TPT	Although this is a clinical standard prior TPT initiation, it is not routinely conducted due to resource constraints. There are a few sentinel sites that are able to conduct tests such as LFTs.	According to HCW 03,<br>*"We do screen the patients but we may not be able to do the relevant tests. We wait for the patient to report the side effects and deal with them accordingly."*<br><br>HCW 09 added that:<br>*"We may not afford to do these LFTs for each and every patient."*

Stockout of TPT drugs	Stockout of TPT drugs was mentioned as one of the recurrent challenges that delays TPT initiation. This was common for pediatric regimens and pyridoxine.	HCW 10 in charge of dispensing TPT drugs said:<br><br>*"Lately, we hardly receive the 25mg tablet, what was supplied is the 50mg tablet and you know pyridoxine they take 25mg, assume it is a child where they have to take 25mg. How do you break the tablet into 2? That tablet is unbreakable, if it happens to break, it will be in very small particles, meaning the drug has been wasted."*
Rigid exclusion criteria for TPT due to clinical trials	There are several studies being conducted whose criteria is different from the national guidelines thus delaying TPT initiation.	According HCW 11,<br><br>*"Interruption by studies on INH and rifapentine has affected TPT uptake"*

Supplementary Table S3: Facilitators for TPT adherence and completion among PLHIV in selected health facilities of Uganda during 2022-2023

**Patient level facilitators for TPT adherence and completion**	These are individual factors that enable and support patients to successfully adhere to and complete their TPT course.	
Perceived benefits of TPT	According to PLHIV, perceived benefits of TPT motivate them to adhere and complete their TPT courses. The benefits mentioned included; reduced risk of TB, enhanced quality of life and improved health.	Patient 03 said that:<br><br>*"The health care workers explained to me that if I take my drugs well, the TPT will prevent me from getting TB"*
Not developing any adverse events	PLHIV are motivated to complete their TPT courses when there aren't any side effects from the drugs.	Patient 04 said:<br><br>*"I didn't get any side effects due to TPT. I was able to take the drugs for all the six months."*
**Provider level facilitators for TPT adherence and completion**	These are factors related to healthcare providers that enable and support patients to successfully adhere to and complete their TPT course.	
Proactiveness of HCWs to visit patients missing TPT refill appointment	Proactiveness of HCWs to conduct community drug refills to ensure continuity of treatment among the patients thus ensuring adherence.	HCW 04 mentioned that:<br><br>"*Community delivery of TPT for patients that fail to keep their appointment, we deliver the drugs to them in the community so that they continue with their course of treatment."*
Ability to offer adherence counselling	Adherence counselling helped patients understand the importance of taking the TPT drugs as prescribed and addresses concerns or barriers that may lead to non-adherence.	HCW 12 mentioned that:<br><br>*"There are also underlying psychosocial issues that affect patients' adherence, so we usually offer psychosocial support to the patients."*
Adherence to SOPs on AE management	Consistent monitoring and effective management of AEs promoted patient safety thereby facilitating TPT adherence and completion. HCWs ensured that patients receive high-quality care, and that	HCW 03 mentioned that:<br><br>*"We make sure that those who are currently taking TPT are evaluated to ensure that they have not developed any TB symptoms. We educate them about adverse*

	AEs are identified and managed promptly and effectively.	*events, like once they see their hands or eyes turning yellow, they report to the HF."*
**System related facilitators for TPT adherence and completion**	These are organizational, structural, and policy-related factors that enable and support patients to successfully adhere to and complete their TPT course.	
Availability of shorter regimens	The availability of shorter TPT regimens addresses the challenge of pill burden among patients. Additionally, it reduces missed appointments as stable patients are given three months' drug refills which doesn't require patients to visit the HF very frequently.	While explaining about 3HP and 1HP, HCW 08 mentioned that:<br><br>*"It is a shorter duration and therefore reduces the pill burden. Patients are relieved of the burden of taking very many pills over an extended period of time."*
DSDM to reduce missed appointments	DSDM facilitates streamlining clinical processes and ensures flexible appointment schedules for both ART and TPT services.	HCW 1 explained that:<br><br>*"The DSDM for ART is integrated with that for TPT. If a patient is given three months of ART (especially the stable clients), we also give them three months for TPT, so that they come to the HF for all the services at once. This way, we are able to reduce on the number of patients who do not complete their TPT course because of missed appointments."*
Linking treatment supporters to improve adherence	Linking patients to treatment supporters enables them to receive comprehensive support including reminders to take the medicines thus improving their ability to adhere to treatment.	 *"There are some stubborn ones who may not adhere even after numerous counselling sessions, we usually attach treatment supporters who will make sure they observe the patient as they take the medicines and ensure that they do not miss any doses."*

Supplementary Table S4: Barriers for TPT adherence and completion among PLHIV in selected health facilities of Uganda during 2022-2023.

**Patient level barriers for TPT adherence and completion**	These are individual factors that hinder patients from successfully adhering to and completing their TPT course.	
Unable to afford food	Due to the dietary requirements, PLHIV are required to have nutritious meals. However, there are some patients that cannot afford proper meals which hinders their adherence to TPT.	HCW 09 said:<br><br>*"Unfavorable home situations such as poverty- families are unable to afford proper meals and therefore cannot adhere to TPT. The current economic situation has made things even worse. There are families that can afford only one meal a day"*
Low priority for consuming TPT	According to HCWs, people fear HIV more than TB hence they prioritize certain medicines over TPT.	HCW 15 explained that:<br><br>*"Due to the pill burden, patients do pill sorting. People tend to respect some drugs and disrespect others. In the long run, you find maybe she left TPT and took ARVs. They try to sort out what suits them best."*
Attrition from ART care	The loss of patients from care during their TPT course hinders adherence and completion. Attrition from care is usually common among migratory populations such as fishermen, long distance truck drivers and sex workers.	HCW 14 said that:<br><br>*"Self-transfer out (usually not documented). Patients shift to other places or change the HF. Even death is inevitable, some patients die. Then there are some that are lost to follow up- usually follow up is done for lost patients, but some patients are untraceable."*
Stigma around TPT	According to the HCs, the stigma around TPT us dues to; knowledge gaps among patients, non-disclosure of HIV status which usually affects appointment keeping.	 *"There is a myth that TPT is for TB patients. Sometimes the patients are stigmatized and they do not want to associate with anything related to TB, as long as you say 'this medicine will help to prevent TB', they do not want to hear about that."*
Missing of appointments	Missed appointments usually due to time or financial constraints are a challenge for drug refills	Patient 05 said:

	which consequently lead to missed TPT doses or treatment discontinuation.	*"Sometimes we have a challenge of transport, and sometimes we may not have the money to come to the hospital."*
Pill burden	TPT adherence is challenging especially among PLHIV that are receiving treatment for other comorbidities such as hypertension, diabetes, among others.	HCW 13 explained that:<br><br>*"Six months is a long time for a tablet every day, in addition to other medicines that the patient has to take (i.e ARVs, anti-hypertensive drugs), and remember we give the TPT with a pyridoxine tablet as well."*
Reluctance among caregivers of children	Reluctance among caregivers of children to administer TPT due to competing priorities can deter adherence and completion.	HCW 16 mentioned that:<br><br>*"When it comes to children, they are dependent on adults to take their medication. So, if the adult does not endeavor to give the medication, this child will not take the drugs."*
**Provider level barriers for TPT adherence and completion**	These are factors related to healthcare providers that hinder patients from successfully adhering to and completing their TPT course.	
Lack of adherence to documentation protocols	All the HFs use TPT registers and the EMR to document TPT initiation, adherence and completion. However, there are inconsistencies in the data sources (i.e EMR vs TPT register and the patient files). For example, the patient file may show that they were initiated on TPT, but without further information on completion.	HCW 17 explained that:<br><br>*"We have some cases of inconsistencies in the information recorded in the patient's card versus the TPT register. Some patients may have been initiated but documentation was not done. Routine file audits have been conducted to triangulate data from all HMIS tools and eliminate such challenges."*
Difficulty in monitoring patient adherence	According to the HCWs, TPT adherence is entirely subjective and dependent on what the patient tells the HCW.	HCW 18s mentioned that:<br><br>"*You just have to believe what the patient tells you. You will never know whether or not they took the TPT. We have tried to count pill balances but it is not an efficient method of ascertain TPT adherence."*

**System related barriers for TPT adherence and completion**	These are organizational, structural, and policy-related factors that hinder patients from successfully adhering to and completing their TPT course.	
Lack of a TPT adherence monitoring tool	TPT adherence can only be ascertained through direct observation of drug intake or counting pill balances, both of which may not be ideal for the HCWs.	HCW 12 explained that:<br><br>*"There are patients who we assume took the TPT but in actual sense, they did not. And we cannot tell because we do not have the routine tests within our setting to tell the drug concentration within the patient are indicative of someone who is currently taking TPT. As you are aware, the guidelines do not provide for that. It is not like viral load which you do to check whether someone is adhering to ARVs. It would have been good if there was that test we could do to tell whether someone is indeed taking TPT."*
Non availability of shorter regimens	TPT adherence and completion is especially poor among children. However, there aren't shorter pediatric TPT regimens other than the 6 months INH.	HCW 01 explained this:<br><br>*"The children should also be considered for shorter TPT regimens because children have the poorest adherence and completion rates."*
Resource constraints for community TPT refills	Due to limited funding, the majority of the programs (such as community TPT refills) are reliant on IP support and donor funding.	HCW 16 mentioned that:<br><br>*"Sometimes they can allocate a resource for a given time and then afterwards, it is withdrawn yet the patients are used to that service. So, I think TB services should be consistent whereby patients can get services whenever needed."*
